# Removal of older males increases extra-pair siring success of yearling males

**DOI:** 10.1371/journal.pbio.3002584

**Published:** 2024-04-16

**Authors:** Emmi Schlicht, Carol Gilsenan, Peter Santema, Agnes Türk, Andrea Wittenzellner, Bart Kempenaers

**Affiliations:** 1 Max Planck Institute for Biological Intelligence, Department of Ornithology, Seewiesen, Germany; 2 Edward Grey Institute, Department of Biology, University of Oxford, Oxford, United Kingdom; The Australian National University, AUSTRALIA

## Abstract

In animals, reproductive performance typically improves over time early in life. Several ultimate and proximate mechanisms may contribute to such an age-related improvement and these mechanisms can act in a relative or in an absolute sense. Low performance of young individuals may be the consequence of a comparison or competition with older individuals (relative), or it may be due to specific traits of young individuals and be unrelated to the presence of older competitors (absolute). Here, we perform a test to disentangle whether the effect of age class (yearling or older) on male extra-pair siring success is relative or absolute. Male age is the most consistent predictor of male extra-pair siring success across bird species, yet the mechanisms underlying this pattern are not well understood. Low extra-pair siring success of yearling males may be a consequence of the presence of older (“adult”) males (hypothesis 1), because adult males are more successful in intra- and intersexual interactions or because females prefer to copulate with adult males when available (relative preference). Alternatively, low extra-pair siring success of yearlings may be independent of the presence of adult males (hypothesis 2), for example, if yearling males on average invest less in extra-pair behavior or if females avoid them as extra-pair mates, independent of the availability of older males (absolute preference). To distinguish between these 2 hypotheses, we experimentally manipulated the age structure of a nest-box-breeding population of blue tits (*Cyanistes caeruleus*) by removing almost all adult males, and compared patterns of extra-pair paternity in the experimental year with those from the preceding 15 “control” years. Removal of adult males resulted in a substantial increase in the extra-pair siring success of yearling males compared to the “control” years, but did not affect the population-level frequency of extra-pair paternity or its spatial patterns. Our results provide clear evidence that extra-pair siring success of yearlings can increase and that it depends on the presence of older males in the population, indicating a relative effect of age on reproductive performance. These results suggest that older males outcompete yearling males in direct or indirect interactions, in sperm competition or as a result of differences in attractiveness to females.

## Abstract 2

Male reproductive performance typically improves over time early in life, but is this due to competition with older males or inherent to younger males? This study of blue tits suggests that older males outcompete yearling males via sperm competition or differences in attractiveness to females.

## Introduction

In many socially monogamous species, some males increase their reproductive success by performing extra-pair copulations and siring one or more offspring in the brood of another pair [1–3] (hereafter referred to as an “extra-pair event” [4,5]). Why some males are more successful than others at siring extra-pair young remains unclear [2], but male age has emerged as the single best predictor of male extra-pair siring success across bird species [6–9]. Typically, yearlings are less likely to sire extra-pair young than adult males, and in all studies that have investigated this, the effect is based on within-individual changes rather than selective disappearance of low-quality individuals [9–12]. However, the mechanism driving the early-life improvement in extra-pair siring success remains unclear [9].

Explanations for why individuals improve their performance with age early in life can be divided into ultimate and proximate ones [13]. On an ultimate level, selection may favor young individuals that perform at a lower level and delay maximum performance until a later age (life-history optimization; [14–16]). Thus, life-history trade-offs may reduce payoffs from extra-pair behavior in yearling males compared to older males. Alternatively, the performance of young individuals may be constrained by selection for ongoing development after attaining independence and reproductive maturity (constrained competence; [13,15]). Under this scenario, yearlings may be unable to attain the extra-pair siring success of adult males, because they have not yet completed all the relevant developmental processes at first breeding. Independent of life-history optimization or developmental constraint, several proximate mechanisms may lead to lower performance of young individuals [13,15,17–19].

- Lack of morphological and physiological maturation. For example, in birds, bone, muscle, and feather development are often still ongoing during an individual’s first breeding season [18,20,21]. A more advanced maturational state of adults compared to yearlings may allow adult males to interfere with extra-pair copulation attempts of yearlings and to prevail in antagonistic interactions as well as in endurance competition (e.g., in their ability to start dawn singing early and maintain singing for longer), leading to a relative disadvantage of yearlings compared to adults. In addition, many birds show delayed plumage maturation [22–24]—potentially including the partial post-juvenile molt of most European passerines ([20], pp. 3 to 4)—and delayed maturation in features of the song [25,26]. Both plumage and song traits are considered important in mediating mating success [27–30]. Similarly, birds show age-related differences in primary reproductive traits (in particular testis size; [31–37]). Finally, birds can also show a delayed onset of reproduction itself, i.e., not reproduce at all in the first year(s) of life [16,38,39]. Threshold effects of this type may impose an absolute limit on the success of yearlings, independent of the social and environmental setting (see, e.g., [24,31]).
- Lack of skills and experience. Competence acquired through learning can influence survival and reproductive performance [40–43], and may explain differences in performance between young and older birds [13,15,17–19]. Adults may have more time and energy available because of their experience (e.g., practice in winter foraging and breeding behavior may improve efficiency and lead to higher energy reserves), which may in turn lead to superiority in interference and endurance competition for extra-pair copulations. Competitive experience (e.g., in fighting) may also positively affect competitive outcomes. Furthermore, experience may influence skills relevant to obtaining extra-pair copulations, such as the ability to form social associations or to identify and locate fertile females. Lack of experience may thus lead to a relative disadvantage in competition and extra-pair mating for yearling males compared to adult males. Alternatively, reduced competence in foraging or other maintenance behaviors [18,19] may affect time and energy budgets of yearlings and shift behavioral trade-offs away from investing in extra-pair behavior. Similarly, reduced mating competences may lead to a failure or lower probability to sire extra-pair offspring. Both would impose absolute constraints on the extra-pair siring success of yearlings, independent of the social setting.
- Reduced access to resources due to the absence of a previous breeding history. Benefits of familiarity (e.g., effects on breeding and mating success; [44–46]) are thought to be a key driver of site fidelity [47]. Prior residency implies less time and energy needed for territory establishment and possibly for pair formation. These effects on time and energy budgets of yearlings may lead to a relative disadvantage in competition over extra-pair copulations or impose an absolute constraint by shifting optimal allocation patterns away from seeking extra-pair matings.
- Lack of attractiveness. Females may prefer older males as copulation partners [22,48–50]. Such a preference may be adaptive if older males provide better resources or are of higher genetic quality [51–56]. Females might also be more responsive to older males if older males are better at convincing or coercing them to perform extra-pair copulations, even if such copulations do not benefit females [8]. In many bird species, females play an active role in extra-pair copulations [57–64]. Thus, low extra-pair success of yearlings may be female-mediated. If a female preference for older over younger males is expressed in a relative sense [65,66], females should only accept yearlings as extra-pair partners when adults are unavailable (relative disadvantage). Alternatively, females may universally reject yearling males as extra-pair partners, independent of the pool of males they can choose from. If the preference for adult males is based on a fixed threshold [65,67–75], yearlings will be excluded as extra-pair mates (absolute exclusion), unless females make assessment mistakes or are deceived into mating with a yearling male while targeting an adult male [63].

Here, we test whether the low extra-pair siring success of yearlings is the consequence of the presence of adult males (hypothesis 1, relative effect) or is independent of the presence of adult males (hypothesis 2, absolute effect). Given that maturational thresholds and female preference thresholds have been previously reported (see above), and that the presence of older individuals can have substantial effects on the performance of yearlings [19,76–81], both hypotheses are plausible. The aim of our study is thus to differentiate between these hypotheses as a first step towards a deeper understanding of the mechanisms underlying the general phenomenon of early-life improvement in reproductive success in the context of avian extra-pair paternity.

To distinguish between the 2 hypotheses, we removed almost all adult males from a population of blue tits (*Cyanistes caeruleus*) in 2022, and we compared the frequency and patterns of extra-pair paternity in the experimental year with data from the same blue tit study population during 15 “control” years (2007 to 2021). Earlier work showed a strong age effect on extra-pair siring success in this species in general [82–85] and also in this particular population [9,86].

We tested predictions from the 2 hypotheses explaining the low extra-pair siring success of yearlings, following a preregistered protocol [87]. First, if low extra-pair siring success of yearlings is due to the presence of adults (hypothesis 1), yearling male breeders should have higher extra-pair siring success in 2022. If low extra-pair siring success of yearlings is independent of the presence of adults (hypothesis 2), the siring success of yearling male breeders should not be affected by the manipulation.

Second, hypothesis 1 predicts that the manipulation should have no effect on the population-level frequency of extra-pair paternity, whereas hypothesis 2 predicts lower levels of extra-pair paternity because males (efficiently) investing in extra-pair behavior or males preferred by females are largely absent.

Third, previous work on the same population showed that most extra-pair sires are direct neighbors, i.e., males breeding on an adjacent territory [88]. However, if females seek extra-pair copulations specifically from adult males, they would need to increase their search radius to obtain these copulations after the manipulation. Similarly, if the investment of yearling males in extra-pair behavior is generally low, the few remaining adult males and those just outside the monitored area could potentially increase the spatial range of their extra-pair activities. The 2 hypotheses thus make different predictions about how the manipulation affects the spatial patterns of extra-pair paternity. Hypothesis 1 predicts no changes in the spatial patterns of extra-pair paternity, whereas hypothesis 2 predicts that extra-pair young should be more often sired by more distantly breeding adult males or by adult males from outside the monitored area.

## Results and discussion

To investigate if the presence of adult males drives the low extra-pair siring success of yearling males, we removed adult males caught in winter and early spring of 2021/2022 such that the 2022 male breeding population consisted almost exclusively of yearlings. Of a total of 75 breeding males in 2022, 71 (95%) were yearlings, whereas in the control years only 26% to 63% of breeding males were yearlings (median: 48%; Fig A.A in S1 Text).

**Fig 1:**
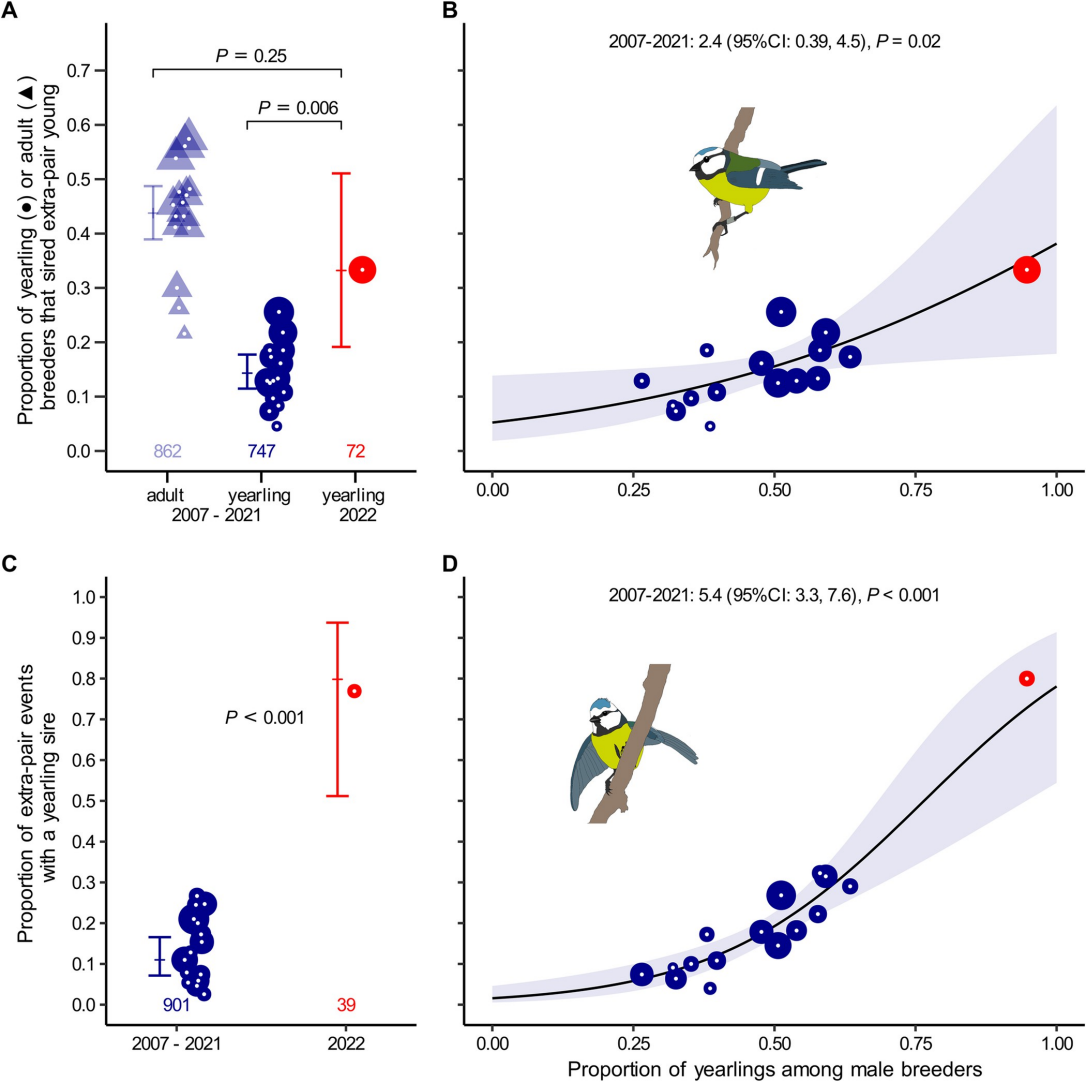
Extra-pair siring success of yearlings was higher in the experimental season (2022) compared to the control seasons (2007–2021). (A) The proportion of yearling male breeders that sired at least 1 extra-pair offspring was significantly higher in 2022 (red) than in the control years (dark blue, circles), and similar to the proportion of adult males that sired extra-pair young in the control years (light blue, triangles). (B) Relationship between the proportion of yearling male breeders that sired at least 1 extra-pair young and the proportion of all breeders that were yearlings in the control years (blue). The data from 2022 (red) fit well with the prediction generated from this relationship. (C) The proportion of all extra-pair events that involved a yearling male sire was significantly higher in 2022 (red) than in the control years (blue). Each extra-pair event represents a unique male–female combination that produced extra-pair young in a given year. (D) Relationship between the proportion of all extra-pair events that involved a yearling male sire and the proportion of breeders that were yearling in the control years (blue). The data from 2022 (red) fit well with the prediction generated from this relationship. Models for (A) and (C) are using a binary response variable (Y/N) at the level of individuals or extra-pair events. Shown are model fits with their 95% confidence intervals (bars or shading) and raw data as annual proportions (symbols, size relative to sample size). Numbers at the bottom indicate overall sample sizes. See Table A in S1 Text for statistical details. The data and code needed to generate this figure can be found in https://osf.io/w7fx6.

In 2022, 33% of yearling male breeders (24 out of 71) sired at least 1 extra-pair offspring, which is significantly higher than in the control years (range: 5% to 26%, median: 13%; Fig 1A and Table A in S1 Text). Furthermore, of all extra-pair sires in 2022, 77% were yearling male breeders (30 of 39 events), which is significantly higher than in the control years (range: 3% to 27%, median: 13%; Fig 1C and Table A in S1 Text). Thus, the extra-pair siring success of yearlings was higher in 2022, when adults were largely absent, than in the control years. These results clearly support hypothesis 1 and show that extra-pair siring success of yearlings is not fundamentally limited by a lack of experience or maturation, by shifts in life-history trade-offs or by a rejection as extra-pair partners by females. Instead, we show that the extra-pair siring success of naïve yearling males increases under conditions of reduced competition with adults.

**Fig 2:**
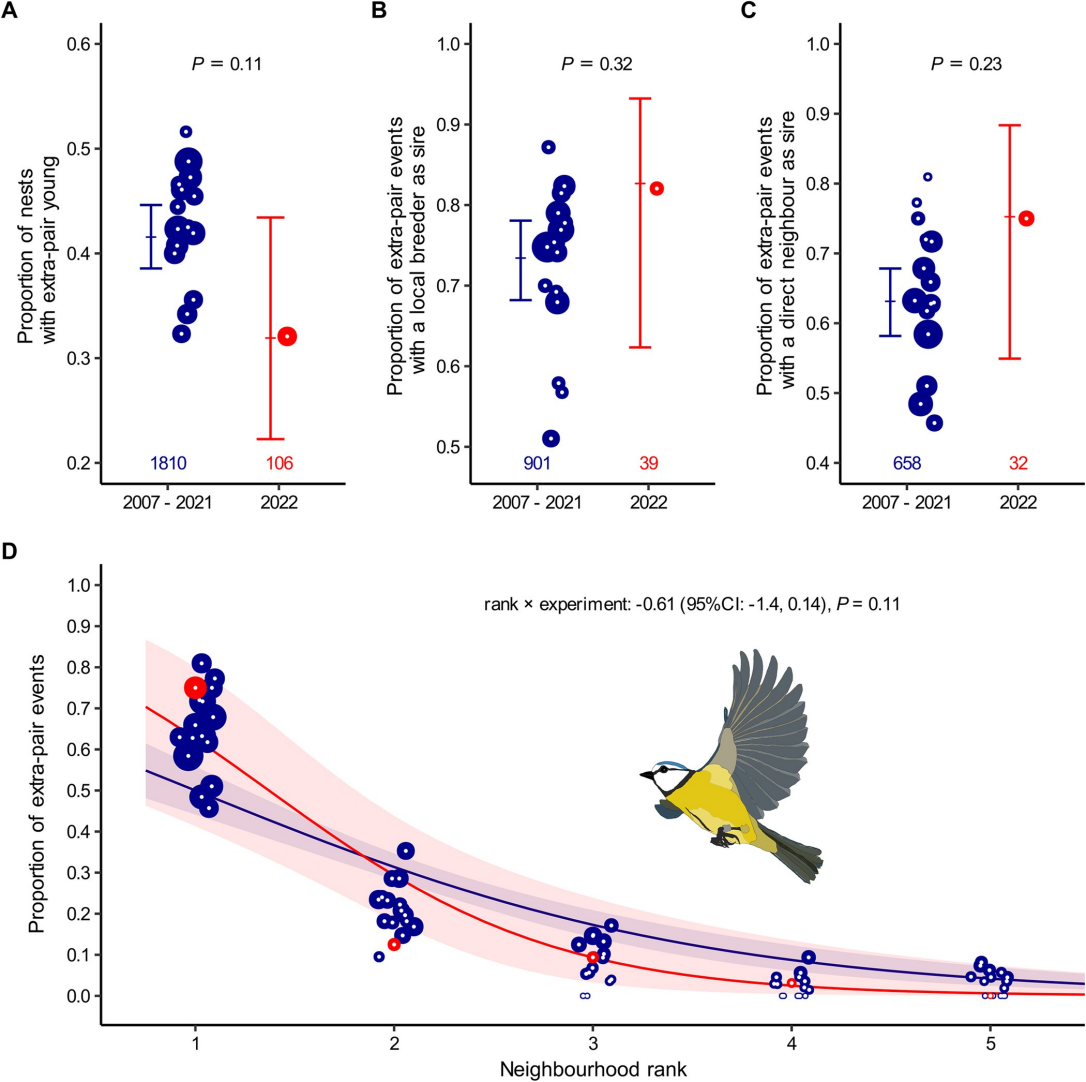
The frequency of extra-pair paternity and its spatial pattern did not differ between the experimental season (2022) and the control seasons (2007–2021). (A) The proportion of nests with extra-pair young. (B) The proportion of extra-pair events with a locally breeding male as a sire. (C) The proportion of extra-pair events, where the sires were locally breeding and had their territory adjacent to the territory where they sired extra-pair young. (D) The distribution of neighborhood ranks for extra-pair events (1 = direct neighbors, 2 = second order neighbors, etc.). All 4 variables did not differ between 2022 (red) and the control years (blue). Each extra-pair event represents a unique male–female combination that produced extra-pair young in a given year. Models for (A) to (C) are using a binary response variable (Y/N) at the level of nests or extra-pair events. Shown are raw data as annual proportions (dots, size relative to sample size) and model fits with their 95% confidence intervals (bars or fitted lines with shaded areas). Numbers at the bottom indicate overall sample sizes. See Table B in S1 Text for statistical details. The data and code needed to generate this figure can be found in https://osf.io/w7fx6.

In further support of hypothesis 1, we found that the proportion of nests with at least 1 extra-pair offspring did not differ statistically between 2022 (33%, 34 of 102 nests) and the control years (median: 43%, range: 33% to 52%; model with binary response variable; Fig 2A and Table B in S1 Text). Thus, the frequency of extra-pair paternity did not clearly decrease in the absence of adult male breeders, indicating that the availability of adult males does not affect the frequency of extra-pair matings and that yearling males “qualify” as extra-pair sires under particular circumstances.

As a result of the manipulation, in 2022 most females had no adult males as a direct neighbor. However, the proportion of extra-pair events that involved a locally breeding male did not statistically differ between 2022 (82%, 32 of 39 events) and the control years (median: 75%, range: 51% to 87%; model with binary response variable; Fig 2B and Table B in S1 Text). Furthermore, the probability that extra-pair young were sired by direct neighbors was similar in 2022 (75%, 24 of 32 events) compared to the control years (median: 63%, range: 46% to 81%; model with binary response variable; Fig 2C and Table B in S1 Text), and the distribution of neighborhood ranks for extra-pair paternity events did not differ between 2022 and the control years (Fig 2D and Table B in S1 Text). Thus, in line with hypothesis 1, the spatial range of extra-pair behavior did not increase when the availability of adult males as extra-pair mating partners was low (female perspective) or when the competition by other adult males was reduced (male perspective). Incidentally, of the 4 adult males that bred on the study site in 2022 only 2 sired extra-pair young, each in a single nest, indicating that the few remaining adult male breeders did not have a disproportionately high extra-pair siring success in 2022.

Thus, both the frequency and spatial patterns of extra-pair paternity were largely unaltered by the manipulation (Fig 2). When few adult males were available, females did not reduce their extra-pair mating rate or extend their search radius within or beyond the study site. Even if extra-pair mating in blue tits is partially mediated by a female preference for adult males, our results suggest that the availability of the preferred adult males did not strongly influence the females’ decisions to engage in extra-pair copulations or the tendency to do so primarily with males that bred nearby.

Our results suggest that the removal of adult males relieved yearling males from direct and indirect effects of the presence of the adults, in line with predictions from hypothesis 1. We would then expect that under natural conditions extra-pair siring success of yearlings will be positively related to the proportion of yearling male breeders in the population. Our data from the control years confirm this, both in terms of the proportion of yearlings that sired extra-pair offspring, and in terms of the proportion of yearlings among all extra-pair sires (Table A in S1 Text). Moreover, the observed extra-pair success of yearlings in 2022 fits well with the success predicted based on these relationships (Fig 1B and 1D), suggesting a causal link between the presence of adult males in the population and the extra-pair siring success of yearlings. In fact, the probability that a yearling male sired extra-pair offspring increased in 2022 to the extent that it reached the probability observed for adult males in the control years (Fig 1A; annual proportion of yearling males that sired extra-pair offspring in 2022: 33%; median annual proportion of adult males that sired extra-pair offspring in the control years: 45%, range: 22% to 57%).

Extra-pair siring success of yearlings may be suppressed in the presence of adult males due to (1) direct interference by adult males; (2) higher investment in extra-pair copulations and related behavior by adult males; (3) higher investment in sperm (numbers or quality) by adult males; or (4) a female preference for adult males. We briefly discuss these 4 possibilities. In many tit species, adult males are dominant over yearlings [89–91] and thus competitively superior in antagonistic interactions. Adult superiority may arise because of morphological build [18,20,21], experience, or prior residency. Thus, under natural conditions adult males may often thwart the extra-pair copulation attempts by yearling males.

During the breeding season, experience and prior residency may allow adult males to save time and energy when performing other tasks such as foraging and territory defense. Adults may then spend more time or energy on behaviors that increase the probability of obtaining extra-pair copulations. Their experience may also lead to higher mating or fertilization success per unit of time or energy spent on such behavior. If persistence or performance relative to other males (e.g., the start of dawn singing [92–94]) influences extra-pair siring success, adult males may prevail compared to yearlings. Additionally, familiarity between opposite sex individuals, i.e., their social network position and associations formed during winter, can influence extra-pair associations in spring (as shown in our population [86,95]. Older males may be able to form associations more easily and thus have higher extra-pair siring success, for example, because they keep associations formed in previous years or because they arrive at the study site earlier in the season [96]. In this context, the vacancies created by the removal of adult males may have enhanced the social network position of yearling males and may have allowed them to gain a territory earlier in the season. In addition, if the presence of adult males increases the threat of losing paternity in the yearling males’ own brood [9], adult removal may have relieved yearling males from having to invest as much in paternity protection behaviors such as territory defense or mate guarding [97], thereby allowing them to invest more in seeking extra-pair copulations. For yearlings, the payoff of investing in extra-pair behavior may thus vary depending on the age composition of the male population, in particular, if paternity loss is age related. Although a higher risk of paternity loss for yearlings compared to adults has been reported in some species [7,9], this is not the case for the blue tit [9].

Adult males may outcompete yearling males in postcopulatory processes. For example, adults may develop larger testes and transfer more sperm or more competitive sperm [31–37,64,98–100]. Thus, even if adult and yearling males have a similar likelihood of obtaining extra-pair copulations, adult males’ copulations may be more likely to lead to fertilizations. However, in our blue tit population, yearling and adult males with the same number of extra-pair mates have similar fertilization success [9]. Nevertheless, we cannot exclude that postcopulatory effects contribute to the age class-related increase in extra-pair siring success.

In the blue tit, females play an active role in extra-pair copulations [57]. If females select or seek extra-pair copulations from the most attractive individuals among the available males in their neighborhood, adult males may be preferred under natural conditions [6,8,9,54,56]. When adults are removed, the likelihood that a yearling male becomes the most attractive extra-pair partner among the available males may increase. Thus, female choice can also explain the observed increase in extra-pair siring success of yearling males. More work is needed to differentiate between these 4 scenarios, but note that they are not mutually exclusive, and all 4 may contribute to the increased extra-pair siring success of yearlings when the population contains fewer adults.

**Fig 3:**
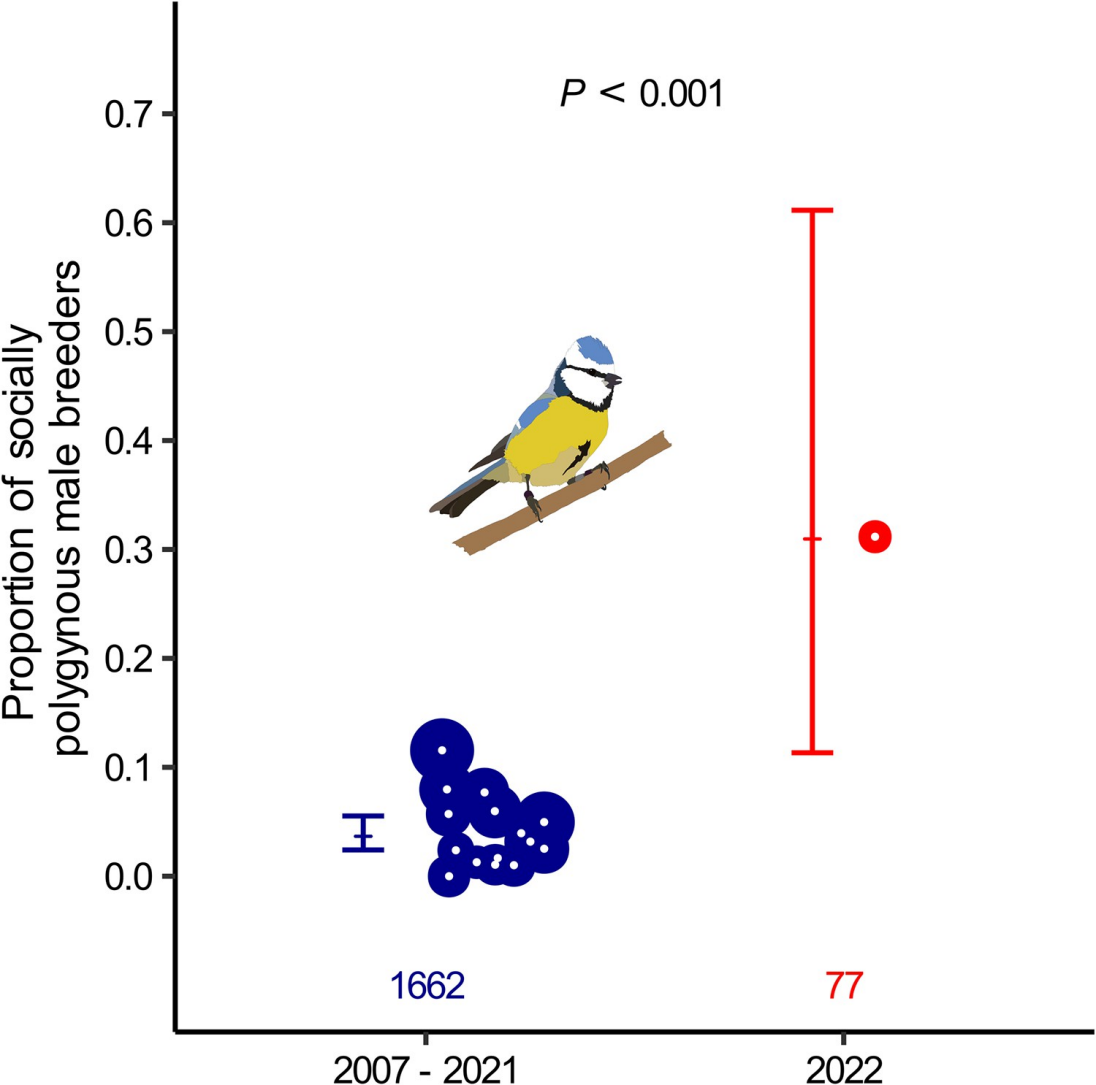
The frequency of social polygyny was higher in the experimental season (2022, red) than in the control seasons (2007–2021, blue). Shown are annual raw data (dots, size relative to sample size) and model fits with their 95% confidence intervals (bars). Numbers at the bottom indicate overall sample sizes. Note that the proportion of socially polygynous males is directly related to the sex ratio of adult breeders, because it defines the excess number of breeding females over breeding males. See Table D in S1 Text for statistical details. The data and code needed to generate this figure can be found in https://osf.io/w7fx6.

Although most breeding parameters did not differ between the 2022 season and the control seasons (Fig A in S1 Text and Table C in S1 Text), we observed an unusually high level of social polygyny in 2022 (Fig 3 and Table D in S1 Text), presumably because our manipulation led to a female-biased population sex ratio. In the control years, the probability of paternity gain increased with the population-level rate of social polygyny (Table E in S1 Text). This suggests that the increase in extra-pair siring success of yearlings in 2022 may be caused by the increase in the frequency of social polygyny instead of the reduced presence of adult breeders. Social polygyny may increase opportunities for extra-pair mating, because in our population paternity loss is higher for socially polygynous males [101]. In another blue tit population, paternity loss was particularly high for socially polygynous males if they were yearlings [102]. Thus, the observed increase in the extra-pair siring success of yearlings in 2022 might also be explained by easier access to extra-pair copulations, either because females of socially polygynous males are more likely to seek copulations with multiple males [101], or because socially polygynous males, in particular yearlings, cannot guard their mates as closely as monogamous males [57,102,103]. Higher extra-pair siring success of yearlings in the experimental year may thus be due to a combination of reduced competition with adults and an increase in opportunities related to the high level of social polygyny. However, both the frequency of social polygyny and the proportion of yearlings in the population had a significant positive effect on extra-pair siring success of yearlings in the control years (Table E in S1 Text), indicating that the age structure of the population affects extra-pair siring success of yearling males regardless of the prevalence of polygyny.

While 2022 did not differ from the preceding years in any other key aspect of blue tits’ reproductive biology (Fig A in S1 Text and Table C in S1 Text), we cannot exclude that 2022 was unusual in some way(s) not measured by us. However, it seems unlikely that this would lead to precisely the effects a priori predicted by one of the 2 hypotheses, as outlined in the preregistration [87].

Improvements in performance early in life, in particular in reproduction, are prevalent throughout the animal kingdom [13,15,104–106] and many explanations may affect performance of young individuals in a relative or an absolute sense (see Introduction). We here perform the critical test to distinguish relative and absolute effects of young age on performance in the context of extra-pair siring success. Our results indicate that the extra-pair siring success of yearling males changed in relation to their social environment. We thus provide clear evidence that low extra-pair siring success of yearling male blue tits is a relative phenomenon.

The distance at which males can sire extra-pair offspring appears to be a less flexible trait, because the manipulation did not increase the spatial range of extra-pair events. The mechanisms that led to a similar spatial structure of extra-pair events in 2022 may fundamentally constrain the plasticity of extra-pair behavior in both males and females. Our study thus confirms the pivotal role of the local neighborhood [88] and established social associations [95] for extra-pair paternity in blue tits.

In conclusion, our study provides clear evidence that the extra-pair siring success of yearling males in blue tits is flexible and not an inevitable consequence of being young, inexperienced, or immature. Removal of adult males from the breeding population (1) released yearling males from direct and indirect effects of competition with adult males; (2) removed opportunities for female choice of extra-pair sires based on age; and (3) may have increased access to extra-pair copulations through an increase in social polygyny. Our study suggests that adult males suppress the extra-pair success of yearling males. If our results are generally valid, they indicate that the age-effect on extra-pair siring success observed across species may be driven by individual variation in competitiveness and attractiveness related to the age structure of the social environment.

## Materials and methods

### Study site

We studied a population of blue tits in a 40-ha oak-rich plot within a mixed-deciduous forest close to Landsberg am Lech, Germany (“Westerholz”, 48°08′26″N 10°53′29″E), from 2007 to 2022. For the purpose of this study, we consider data from the 2007 to 2021 breeding seasons the natural control and data from the 2022 breeding season the experimental treatment.

The study area contained 277 nest-boxes permanently equipped with an RFID antenna around the entrance hole and with 2 light barriers (one on the outside and one on the inside [107]). Each blue tit carrying a transponder is automatically recorded when it passes through the nest hole, with data on bird identity, date, time, and—based on information from the light barriers—box entry or exit.

### Study species

Blue tits are small (ca. 10 to 12 g) cavity-nesting passerines that inhabit all-purpose territories, which they defend with increasing intensity from early spring onwards [108]. Overwinter survival is low [108,109]. In our population, 42% of breeders are observed again in a later year (median [range] across 2007 to 2021: 45% [19% to 58%]). In any given year on average (± SD) 46% ± 11% of male breeders are yearlings (range across 2007 to 2021: 26% to 63%; Fig A.A in S1 Text). Blue tits are socially monogamous with occasional social polygyny [101] and frequent extra-pair paternity [83–85,102,110,111]. In our population, 43% of nests contain at least 1 extra-pair young (median [range] of annual proportion 2007 to 2021: 43% [33% to 52%]; Fig 2A) and 11% of offspring are extra-pair (median [range] of annual proportion 2007 to 2021: 11% [8% to 14%]; see Table F in S1 Text for basic metadata and contextual information regarding parentage). Median clutch size is 10 (range 4 to 16) with little annual variation (median [range] of annual averages: 9.5 [8.6 to 11.1]; Fig A.H in S1 Text). In our study area, blue tits breed between March and June (nest building to fledging). Individuals may produce a replacement brood if the first breeding attempt fails, but there are no second broods (i.e., additional breeding attempts after fledging of a first brood). After fledging in May to June, fledglings perform a partial post-juvenile molt between July and October [20]. These yearlings may then settle on their own territory starting in autumn and can first reproduce the following spring [108].

### Field procedures

Each year, we monitored breeding activity in all nest-boxes from the first start of nest building in early March until the failure or fledging of all nests by the end of June. We visited every nest-box at least weekly (daily around egg laying, hatching, and fledging) to record the stage of nest building, the start of laying (laying date), clutch size, and the date of hatching and fledging. We banded nestlings and took a blood sample when they were 13 to 15 days old.

We captured blue tits throughout the year, either at the nest-box when they fed nestlings (majority of captures until winter 2014) or with a mist net between September and March (majority of captures since winter 2014). A few birds were caught with a snap trap or when they roosted in a nest-box. Each individual was banded, measured, sexed based on plumage coloration and/or reproductive morphology (brood patch, cloacal protuberance), and aged based on plumage characteristics (partial post-juvenile molt [20,112]) as yearling (age = 1 year) or adult (age >1 year). We also took a 5 to 10 μl blood sample from the brachial vein and implanted a transponder under the skin on the back. For captures performed in the 2021/2022 season, the age of all birds handled before September 2021 or hatched in nests monitored on the study site was verified based on information from previous years of the long-term study. Sex of all captured birds was verified via genotyping.

We used a combination of information to identify the social parents of a nest: (1) observations of individuals defending the nest-box or feeding nestlings; (2) nest-box visits based on the transponder data; (3) individuals caught at the nest-box during nestling feeding; and (4) parentage information. When parentage information was used to verify the identity of a male breeder, this was done blind with respect to male characteristics (including age). A male was defined as socially polygynous when he was assigned as the social father to more than 1 nest and egg laying in the later nest started before the earlier nest had fledged or failed [101].

### Laboratory procedures

For molecular sex determination and parentage analysis, we extracted DNA from all blood samples and—when possible—from embryo or nestling tissue. We genotyped each individual using 14 microsatellite markers as described in [101]. We compared the genotypes of parents and their offspring using the software CERVUS [113]. In some cases, offspring could not be assigned to any of the captured individuals (based on likelihood assignment in CERVUS), either because an extra-pair father was not sampled (203 young from 122 broods, 8% of extra-pair young), or because of failure to capture the social parents at a nest (107 young from 43 broods, none in 2022). The probability of erroneously excluding the social male as sire was small (<0.32 × 10^−6^ in all years for the second parent, given that the mother was known; no intraspecific brood parasitism has been detected in the study area). In all years, parentage analysis was conducted blind with respect to male characteristics (including age), and in 2022, we confirmed that none of the translocated males sired offspring by including them as candidate sires.

In 2022, all local male breeders were genotyped, but 4 extra-pair young in 3 nests were sired by unsampled males. The inferred paternal genotype from these young suggested that 3 different males sired the young in these 3 nests. We therefore included 3 extra-pair events with different unknown extra-pair sires for 2022 when comparing the probability that extra-pair sires were breeding locally.

### Translocation

In winter and early spring 2021/2022, we removed 184 adult individuals from the population and released them at least 30 km away from the study site. Almost all (97%) of these individuals were males. The majority (80%) was translocated in winter and the last translocation was performed 14 days before the start of egg laying in a focal territory. Only a few adult females were translocated and the age structure of female breeders remained unaltered in 2022 when compared to the control years (Fig A.B in S1 Text and Table C in S1 Text). Six individuals returned to the study site after translocation (5 males, 3 of them were translocated a second time and did not return, and 1 female). Except for the female, none of these individuals bred in the study site in 2022. The translocations resulted in a 2022 breeding population where 95% of all male breeders were yearlings (71 out of 75).

This study is not an experiment sensu strictu, because individuals were not randomly assigned to a control and a treatment group. Instead, we performed a field manipulation in one particular year and compared the results with those of the previous 15 years. To establish that the 2022 breeding season is comparable with previous breeding seasons, we evaluated how similar the data set of 2022 is to the data from 2007 to 2021 in key aspects of blue tit biology (Fig A in S1 Text and Table C in S1 Text).

### Ethical note

All procedures we implemented are standard ornithological field procedures [114–117]. For a more detailed description of ethical implications, see [118,119]. Permits for the long-term study (permit numbers 55.1–8642.3-7-2006, 55.2-1-54-2532-73-2016, ROB-55.1-8646.NAT_02-6-19-8, and ROB-55.2-2532.Vet_02-21-119) and the translocation experiment (permit number ROB-55.1-8646.NAT_02-6-28-3) were obtained from the Bavarian government and the Bavarian regional office for forestry (LWF, permit number NL360-2006-5708-gul).

To maximize the objectivity of our research, we preregistered the hypotheses and methods of this study before the start of the 2022 breeding season [87]. We closely adhered to our research plan.

### Statistical models

We used linear models (LMs), generalized linear models (GLMs), linear mixed models (LMMs), and generalized linear mixed models (GLMMs) in the statistical software R version 4.2.1 [120]. For all but one (see below) mixed model we used the package “lme4” version 1.1.30 [121] in combination with the package “lmerTest” version 3.1.3 [122]. Model assumptions were checked using the package “DHARMa” version 0.4.6 [123]. The key explanatory variable in most models was the experimental treatment (data from 2022) versus the control (data from 2007 to 2021). Covariates and random intercepts were included as detailed below. GLMMs with binomial error structure use the logit link function. We used the package “ggplot2” version 3.4.0 for making the figures [124].

We evaluated population-level differences in breeding parameters between the 2022 season and the control seasons (Table C in S1 Text, model details in table). We examined breeding density (the number of breeding pairs, i.e., unique male–female combinations), breeding synchrony (calculated following [125]), laying date, clutch size, the proportion of a clutch that hatched, and the proportion of nests that produced ≥1 fledgling. For females, we compared the number of breeding females, the probability that female breeders were yearlings, the probability that breeding females had bred in the previous season (breeders with local breeding experience that survived from the previous season), the probability that breeding females were first recorded in the present season (immigrants), and the probability that breeding females hatched on the study site in the previous season (local recruits). Female survivors, immigrants, and local recruits were inspected to examine any effects of the manipulation on overall population dynamics. For males, we verified that the manipulation had the desired effect on the proportion of yearlings among breeding males (Table C in S1 Text) and examined the effect of the manipulation on the proportion of breeding males that were socially polygynous (Table D in S1 Text) and the number of breeding males (Table C in S1 Text). We did not consider the survivors, immigrants, and local recruits for males, because these were obviously altered as part of the manipulation.

To test whether extra-pair success of yearling male breeders had changed in 2022, we ran 2 binomial GLMMs (Table A in S1 Text). In the first model, the dependent variable was the proportion of yearling male breeders that sired at least 1 extra-pair young, i.e., whether a yearling male breeder in a given year was an extra-pair sire (yes/no). In the second model, the dependent variable was the proportion of yearling male breeders among all extra-pair sires, i.e., whether a sire of an extra-pair offspring was a yearling male breeder (yes/no), whereby we considered each unique male–female combination that produced extra-pair young in a given year as a separate extra-pair event (i.e., grouping multiple extra-pair young by the same sire in the same brood into a single data point). We included study year and nest identity as random intercepts.

To test whether the frequency of extra-pair paternity had changed in 2022, we used a binomial GLMM to compare the proportion of nests that contained at least 1 extra-pair young between the experimental year and the control years (Table B in S1 Text). The dependent variable was the presence of extra-pair young (yes/no) in a nest, and we included study year and female identity as random intercepts. To account for the possibility that the probability of detecting extra-pair paternity is higher for larger clutches [126–128], we also included clutch size as a covariate.

To test whether the distance between the own nest of the extra-pair sire and the nest where he sired the extra-pair young had changed in 2022, we ran 2 analyses (Table B in S1 Text).

First, we used a binomial GLMM to compare the proportion of extra-pair sires that did not breed in the study site (i.e., sires with no brood assigned among the monitored nests in the focal year, including previously captured and unknown males). The response variable was whether an extra-pair sire was a local breeder (yes/no), whereby we considered all unique male–female combinations that produced extra-pair young in a given year as the sample. We included study year and nest identity as random intercepts. Second, for extra-pair events involving locally breeding sires, we compared the distance between the nest of the extra-pair sire and the nest where he sired extra-pair young as the “neighborhood distance” [88], i.e., we assigned territory boundaries by calculating Thiessen polygons [129] and computed the neighborhood rank (i.e., direct neighbors = 1, second order neighbors = 2, etc.).

We compared neighborhood rank in 2 models. (1) We modeled whether the extra-pair sire was a direct neighbor (neighborhood rank = 1, yes/no) with a binomial GLMM. We used all unique male–female combinations that produced extra-pair young in a given year and involved a locally breeding male as a sire. Study year, sire identity, and nest identity were included as random intercepts. (2) For the same data, we compared the distribution of neighborhood ranks between 2022 and the control years with a GLMM fitted with package “glmmTMB,” version 1.1.4 [130]. As the response variable, we used the extra-pair events (i.e., the unique male–female combinations that produced extra-pair young in a given year and involved a locally breeding male as sire) and calculated for each year which proportion of events had a given neighborhood rank (Fig 1D). We assumed an error structure based on the beta distribution with a logit link and included study year as random intercept. We then tested for an interaction effect of neighborhood rank and treatment, i.e., experimental (2022) versus control (2007 to 2021).

Note that we did not run these models for the subset of extra-pair events that involved locally breeding adult sires only, because the sample size in 2022 was too small (*N* = 2).

### Data subsets and additional tests

We examined the effects of excluding subsets of data that could be considered outliers on our results. These included small clutches, replacement breeding attempts, and clutches with incomplete sampling. In our population, 97% of clutches are larger than 4 eggs. When excluding clutches smaller than 5 eggs (*N* = 52), results remained unchanged (for details, see [131]) except for the effect of clutch size on the frequency of extra-pair paternity, which was absent when excluding these unusually small clutches. Because this relationship was not the target of our analysis, and because some of these clutches contained extra-pair offspring, we report the results including all clutches.

Replacement attempts sometimes occur when a clutch fails during the laying, incubation, or early-nestling period. Individuals may then start a second breeding attempt with the same mate (if still alive) or with a new mate. We used 2 criteria to inspect potential confounding effects of replacement nests: (1) known replacement nests, defined as breeding events where we recorded a prior failed breeding attempt for 1 or both social parents; and (2) late nests, defined as all breeding events with a laying date more than 2 standard deviations after the annual mean laying date, which includes 50% of the known replacement nests (*N* = 139). This assumes that the original attempt for these late breeders remained unobserved, because it occurred outside the study area or in a natural cavity. Excluding data from either known replacement nests only or from both known replacement nests and late nests did not affect our results (for details, see [131]). Because some of these nests included extra-pair young, we here present the data including all breeding attempts.

Our sampling of offspring is incomplete, because (1) some broods were depredated; (2) some unhatched eggs and dead chicks did not yield DNA of sufficient quality; and (3) some dead hatchlings were removed from the nest by one of the parents before they could be collected. The median number of completely missing clutches across the 16 study years was 2 (range: 0 to 15). For clutches that were not missing completely, the average (± SD) proportion of the clutch that could be genotyped was 90% ± 14% (range: 9% to 100%; *N* = 1,843 clutches).

The proportion of clutches without any genotyped offspring did not differ between 2022 and control years (binomial GLM: *P* = 0.29, *N* = 1,903 breeding attempts, of which 51 did not lead to any genotyped young, one of them in 2022). Similarly, the proportion of a clutch that was genotyped did not differ between 2022 (88% ± 0.4%, *N* = 103) and the control years (88% ± 0.5%, *N* = 1,894; binomial GLMM with year as random factor: *P* = 0.39).

When we excluded data from clutches for which less than 70% of the eggs had been genotyped (136 or 7% of nests and 686 or 4% of offspring), all results remained unaltered, both qualitatively and quantitatively (for details, see [131]). When we excluded data from clutches for which less than 90% of the eggs had been genotyped (614 or 33% of nests and 4,347 or 27% of offspring), all results remained qualitatively unaltered (for details, see [131]), but effect sizes changed for 2 models (proportion of yearlings among extra-pair sires and proportion of adjacent events for locally breeding adult extra-pair sires) in combination with issues of convergence and model fit, suggesting that these 2 data sets became critically small. Because only 52% of nests (*N* = 960 nests, consisting of 8,949 or 56% of offspring) are completely genotyped, sample sizes for analyses based on fully sampled nests alone were too small (i.e., issues of convergence and model fit for many tests). Nevertheless, these analyses suggest that our conclusions are not affected by incomplete sampling of offspring. We here present the results based on data from all nests to avoid exclusion of nests with extra-pair offspring.

Social polygyny (i.e., a male forming a social bond with 2 or 3 females, see [101]) occurred more frequently in 2022 (31% of all male breeders) than in the control years (range: 0% to 12% of male breeders; Fig 3 and Table D in S1 Text). The most plausible explanation is that the early removal of many males depleted the buffer of males that could have filled vacancies, thereby creating a female-biased population sex ratio and additional opportunities for males to form social bonds with multiple females. We therefore also examined whether the occurrence of social polygyny in 2022 affected our conclusions. Thus, in all models using male breeders, we included whether the male was socially polygynous or monogamous. In models using a sample of nests, we included whether the nest was from a socially polygynous or monogamous male. We included the variable “social polygyny” (yes/no) in interaction with the main effect (experimental year versus control years). This interaction was not significant in any model (for details, see [131]), indicating that patterns of extra-pair paternity were not substantially affected by the occurrence of social polygyny in 2022.

## Supporting information

S1 Text Document containing supporting information (Tables A–F and Fig A). (PDF)

## Abbreviations

GLM: generalized linear model
GLMM: generalized linear mixed model
LM: linear model
LMM: linear mixed model
