# Supplementary material for: Removal of older males increases extra-pair siring success of yearling males
Source: PLoS Biol. 2024 Apr 16;22(4):e3002584. doi: 10.1371/journal.pbio.3002584 (PMC11020368; doi:10.1371/journal.pbio.3002584)
Supplement: S1 Text — (PDF) [file pbio.3002584.s001.pdf]

S1 Text
SUPPORTING INFORMATION

for

Removal of older males increases extra-pair siring success of yearling males

Emmi Schlicht, Carol Gilsenan, Peter Santema, Agnes Türk, Andrea Wittenzellner, Bart Kempenaers*

* bart.kempenaers@bi.mpg.de

This supporting information (S1 Text) contains

- Table A. Extra-pair siring success of yearlings in 2022 compared to 2007 – 2021.
- Table B. Frequency of extra-pair paternity and its spatial pattern in 2022 compared to 2007 – 2021.
- Table C. Comparison of breeding parameters between 2022 and the control years 2007 – 2021.
- Table D. Comparison of the frequency of social polygyny between 2022 and the control years 2007 – 2021.
- Table E. The effects of the proportion of yearlings among male breeders and the proportion of socially polygynous males on extra-pair siring success of yearlings in the control years 2007-2021.
- Table F. Basic parentage metadata and contextual information for the present study.
- Fig A. Comparison of key breeding parameters between the experimental year 2022 and the control years 2007 – 2021.
- Supporting References.

SUPPORTING TABLES

Table A. Extra-pair siring success of yearlings in 2022 compared to 2007 – 2021

Model	Effect	Term (fixed) or group (random)	Estimate (fixed) or SD (random)	Lower 95% CI	Upper 95% CI	*z*	*P*
Probability for yearlings or adults to sire extra-pair young[a]	fixed	(intercept)	-0.699	-1.49	0.0810		
		yearlings 2007-2021 vs. yearlings 2022	-1.09	-1.93	-0.274	-2.7	0.006
		adults 2007-2021 vs. yearlings 2022	0.448	-0.364	1.26	1.1	0.25
	random	year	0.283				
Annual proportion of yearlings that sired extra-pair young[b]	-	(intercept)	-2.90	-4.01	-1.86		
		annual proportion of yearlings among male breeders	2.42	0.387	4.54	2.3	0.02
Probability that extra-pair event involved a yearling sire[c]	fixed	(intercept)	-2.11	-10.6	-1.67		
		2022 vs. 2007-2021	3.49	2.22	20.8	4.6	< 0.0001
	random	year	0.519				
		nest ID	0.718				
Annual proportion of yearling sires among extra-pair events[b]	-	(intercept)	-4.14	-5.29	-3.08		
		annual proportion of yearlings among male breeders	5.41	3.31	7.63	4.9	< 0.0001

[a] Binomial GLMM (estimates on logit scale, binary response variable), study year included as random intercept. N = 1681 male breeders across 16 years.

[b] Binomial GLM (estimates on logit scale), N = 15 years.

[c] Binomial GLMM (estimates on logit scale, binary response variable), study year and nest identity included as random intercepts. N = 940 male-female combinations as genetic parents of extra-pair offspring from 808 nests across 16 years.

Table B. Frequency of extra-pair paternity and its spatial pattern in 2022 compared to 2007 – 2021.

Model	Effect	Term (fixed) or group (random)	Estimate (fixed) or SD (random)	Lower 95% CI	Upper 95% CI	*z*	*P*
Probability that a nest contained extra-pair young[a]	fixed	(intercept)	-1.17	-1.66	-0.659		
		2022 vs. 2007-2021	-0.414	-0.905	0.0853	-1.6	0.11
		clutch size	0.0881	0.0353	0.137	3.4	0.0007[b]
	random	year	0.103				
		female ID	0.687				
Probability that an extra-pair event involved a locally breeding sire[c]	fixed	(intercept)	1.02	0.815	1.26		
		2022 vs. 2007-2021	0.536	-0.493	2.09	1.0	0.33
	random	year	0.339				
		nest ID	0.181				
Probability that an extra-pair event (locally breeding sires) involved a direct neighbor as sire[d]	fixed	(intercept)	0.538	0.314	0.762		
		2022 vs. 2007-2021	0.574	-0.299	1.76	1.2	0.23
	random	year	0.215				
		sire ID	< 0.0001				
		nest ID	0.237				
Relationship between the proportion of extra-pair events and neighborhood rank[e]	fixed	(intercept)	0.782	0.398	1.17		
		rank	-0.782	-0.958	-0.606	-8.7	< 0.0001
		2022 vs. 2007-2021	1.13	2.64	-0.376	1.5	0.14
		rank × (2022 vs. 2007-2021)	-0.613	-1.37	0.142	-1.6	0.11
	random	year	< 0.0001				

[a] Binomial GLMM (estimates on logit scale, binary response variable), study year and female identity included as random intercepts. N = 1889 nests of 1120 females.

[b] Effect of clutch size driven by abnormally small clutches and absent when excluding clutches < 5 (see methods).

[c] Binomial GLMM (estimates on logit scale, binary response variable), study year and nest identity included as random intercepts. N = 938 male-female combinations as genetic parents of extra-pair young from 807 nests across 16 years. Overall, only six extra-pair sires were not breeding locally in 2022: one yearling male (sire at two locations), two adult males and three unknown males.

[d] Binomial GLMM (estimates on logit scale, binary response variable), study year, sire identity and nest identity included as random intercepts. N = 690 male-
female combinations as genetic parents of extra-pair young from 246 nests involving 371 sires across 16 years.

[e] Beta GLMM (estimates on logit scale), study year included as random intercept. N = 74 proportions of rank values across 16 years.

Table C. Comparison of breeding parameters between 2022 and the control years 2007 – 2021.

Model	Effect	Term (fixed) or group (random)	Estimate (fixed) or SD (random)	Lower 95% CI	Upper 95% CI	Statistic	*P*
Probability that a male is a yearling[a]	fixed	(Intercept)	-0.177	-0.430	0.0650		
		2022 vs. 2007-2021	3.11	1.83	4.55	*z* = 4.6	< 0.0001
	random	year	0.412				
		male ID	< 0.0001				
Probability that a female is a yearling[b]	fixed	(intercept)	-0.100	-0.355	0.125		
		2022 vs. 2007-2021	-0.880	-1.93	0.119	*z* = -1.7	0.08
	random	year	0.438				
		female ID	0.166				
Number of breeding males[c]	-	(intercept)	110	93	128		
		2022 vs. 2007-2021	-35	-106	36	*t* = -1.0	0.31
Number of breeding females[d]	-	(intercept)	117	96	138		
		2022 vs. 2007-2021	-15	-98	68	*t* = -0.4	0.71
Breeding density[e]	-	(intercept)	120	98	142		
		2022 vs. 2007-2021	-15	-103	73	*t* = -0.4	0.72
Breeding synchrony[f]	-	(intercept)	0.66	0.60	0.72		
		2022 vs. 2007-2021	0.079	-0.11	0.25	*t* = 0.8	0.47
Laying date[g]	fixed	(intercept)	108	106	111		
		2022 vs. 2007-2021	0.56	-9.6	11	*t* = 0.1	0.92
	random	year	5.0				
		female ID	1.5				
		residual	6.7				
Clutch size[h]	fixed	(intercept)	9.8	9.4	10.2		
		2022 vs. 2007-2021	-0.80	-2.4	0.83	*t* = -1.0	0.36
		female age class (adult vs. yearling)	0.21	0.061	0.37	*t* = 2.7	0.006
		laying date (centralized)	-0.17	-0.13	-0.11	*t* = -20	< 0.0001
	random	year	0.80				
		female ID	1.1				
		residual	1.4				
Number hatched[i]	fixed	(intercept)	0.752	0.650	0.852		
		2022 vs. 2007-2021	0.0343	-0.146	0.215	*z* = 0.4	0.69
		clutch size	0.135	0.126	0.144	*z* = 29	< 0.0001
	random	year	0.0762				
		female ID	< 0.0001				
Fledging success[j]	fixed	(intercept)	1.39	1.16	1.66		
		2022 vs. 2007-2021	-0.257	-1.19	0.870	*z* = -0.5	0.60
	random	year	0.417				
		female ID	0.0713				
Probability that a female had local breeding experience[k]	fixed	(intercept)	-0.464	-0.707	-0.224		
		2022 vs. 2008-2021	0.628	-0.254	1.51	*z* = 1.5	0.14
	random	year	0.352				
		female ID	0.266				

Table C (continued).

Model	Effect	Term (fixed) or group (random)	Estimate (fixed) or SD (random)	Lower 95% CI	Upper 95% CI	Statistic	*P*
Probability that a female was an immigrant[l]	fixed	(intercept)	0.417	0.167	0.670		
		2022 vs. 2008-2021	-0.707	-1.63	0.217	*z* = -1.6	0.11
	random	year	0.373				
		female ID	0.290				
Probability that a female was a local recruit[m]	fixed	(intercept)	-1.60	-1.91	-1.33		
		2022 vs. 2008-2021	-0.435	-1.58	0.71	*z* = -0.8	0.43
	random	year	0.434				
		female ID	< 0.0001				

[a] The probability that a male breeder is a yearling. Binomial GLMM (estimates on logit scale, binary response variable) with year and male identity as random intercepts. N = 1685 male breeders across 16 years (2007 to 2022).

[b] The probability that a female breeder is a yearling. Binomial GLMM (estimates on logit scale, binary response variable) with year and female identity as random intercepts. N = 1798 observations of 1071 females across 16 years (2007 to 2022). In 2022, yearling females had a similar probability to have extra-pair young in their nest (36 %) as adult females (30%; *P* = 0.58; see [S1]). This was also true in control years [S2].

[c] Linear model (LM); N = 16 years (2007 to 2022).

[d] LM; N = 16 years (2007 to 2022).

[e] Number of breeding pairs, i.e. unique male-female combinations; LM; N = 16 years (2007 to 2022).

[f] Breeding synchrony index [S3]; LM; N = 16 years (2007 to 2022).

[g] Laying date measured as day of year (1 = 01. January). Linear mixed model (LMM) with year and female identity as random intercepts. N = 1898 clutches of 1120 females across 16 years (2007 to 2022).

[h] LMM with female age class (yearling or adult) and laying date (centralized within year) as covariates and study year and female identity as random intercepts. A model without the covariates gave similar results (not shown). N = 1847 clutches of 1071 females across 16 years (2007 to 2022).

[i] Poisson GLMM (estimates on log scale) with clutch size as covariate and year and female identity as random intercepts. N = 1877 broods of 1110 females across 16 years (2007 to 2022).

[j] Probability that a given nest produced at least one fledgling. Binomial GLMM (estimates on logit scale, binary response variable) with year and female identity as random intercepts. N = 1900 broods of 1122 females across 16 years (2007 to 2022).

[k] The probability that a female breeder was breeding in the previous season. Binomial GLMM (estimates on logit scale, binary response variable) with year and female identity as random intercepts. N = 1772 observations of 1081 females across 15 years (2008 to 2022; 2007 excluded since no data from previous year available).

[l] The probability that a female breeder was first recorded in the focal season. Binomial GLMM (estimates on logit scale, binary response variable) with year and female identity as random intercepts. N = 1772 observations of 1081 females across 15 years (2008 to 2022; 2007 excluded since no data from previous year available).

[m] The probability that a female breeder hatched from a nest on the study site in the previous season. Binomial GLMM (estimates on logit scale, binary response variable) with year and female identity as random intercepts. N = 1772 observations of 1081 females across 15 years (2008 to 2022; 2007 excluded since no data from previous year available).

There is no relationship between population-wide measures of timing of breeding (mean annual lay date or mean annual hatch date) and the rate of extra-pair paternity for all years (2007-2022) or only during the control years (all P>0.54; see [S1]).

There is no relationship between lay date, hatch date, hatching success or fledging success and the occurrence of extra-pair offspring in a nest and there is no interaction with experimental year (all *P*>0.12; see [S1]).

Table D. Comparison of the frequency of social polygyny between 2022 and the control years 2007 – 2021.

Effect	Term (fixed) or group (random)	Estimate (fixed) or SD (random)	Lower 95% CI	Upper 95% CI	*z*	*P*
Fixed	(intercept)	-3.27	-3.80	-2.90		
	2022 vs. 2007-2021	2.48	1.06	4.07	3.7	0.0002
Random	year	0.587				

The probability that a male breeder is socially polygynous. Binomial GLMM (estimates on logit scale, binary response variable) with year as random intercept. N = 1735 breeding males across 16 years (2007 to 2022).

Table E. The effects of the proportion of yearlings among male breeders and the proportion of socially polygynous males on extra-pair siring success of yearlings in the control years 2007-2021.

Response	Term (fixed) or group (random)	Estimate (fixed) or SD (random)	Lower 95% CI	Upper 95% CI	*z*	*P*
Annual proportion of yearlings that sired extra-pair young	(intercept)	-3.12	-3.89	-2.39		
	proportion yearling male breeders	2.04	0.623	3.51	2.8	0.02
	proportion socially polygynous male breeders	7.82	3.90	11.7	3.9	0.002
Annual proportion of yearling sires among extra-pair events	(intercept)	-4.28	-5.46	-3.19		
	proportion yearling male breeders	5.19	3.05	7.45	4.6	< 0.0001
	proportion socially polygynous male breeders	4.64	-1.32	10.5	1.5	0.12

Binomial GLMs (estimates on logit scale). N = 15 years (2007-2021). There is only a weak correlation between the proportion of yearling male breeders and the proportion of polygynous male breeders (Spearman rank correlation: r_S = 0.15, N = 15, *P* = 0.59) and there is no interaction between the two effects (*P* = 0.38, see [S1]).

Table F. Basic parentage metadata and contextual information for the present study[a].

Location of study population	latitude: 48°08′26″N, longitude: 10°53′29″E
Time of study	control years: 2007 – 2021<br>experimental year: 2022
Social context of families	social monogamy with facultative social polygyny
Basic metadata[b]	

	control years		experimental year	
	social monogamy	social polygyny	social monogamy	social polygyny
number of families sampled	1607	158	55	51
number of broods sampled	1643	159	55	51
number of offspring sampled	14092	1153	452	374
number of offspring found to be within-pair	12439[c]	933[c]	418	318
number of offspring found to be extra-pair	1493[c]	205[c]	34	56
number of broods that contained extra-pair young	688	86	18	16

[a] Information following [S4].

[b] Table shows data from all monitored nests. Models may include smaller sample, if some information is unavailable. See [S1] for data selection for each model.

[c] Difference to total number of offspring sampled explained by young with unassigned paternity status.

SUPPORTING FIGURES

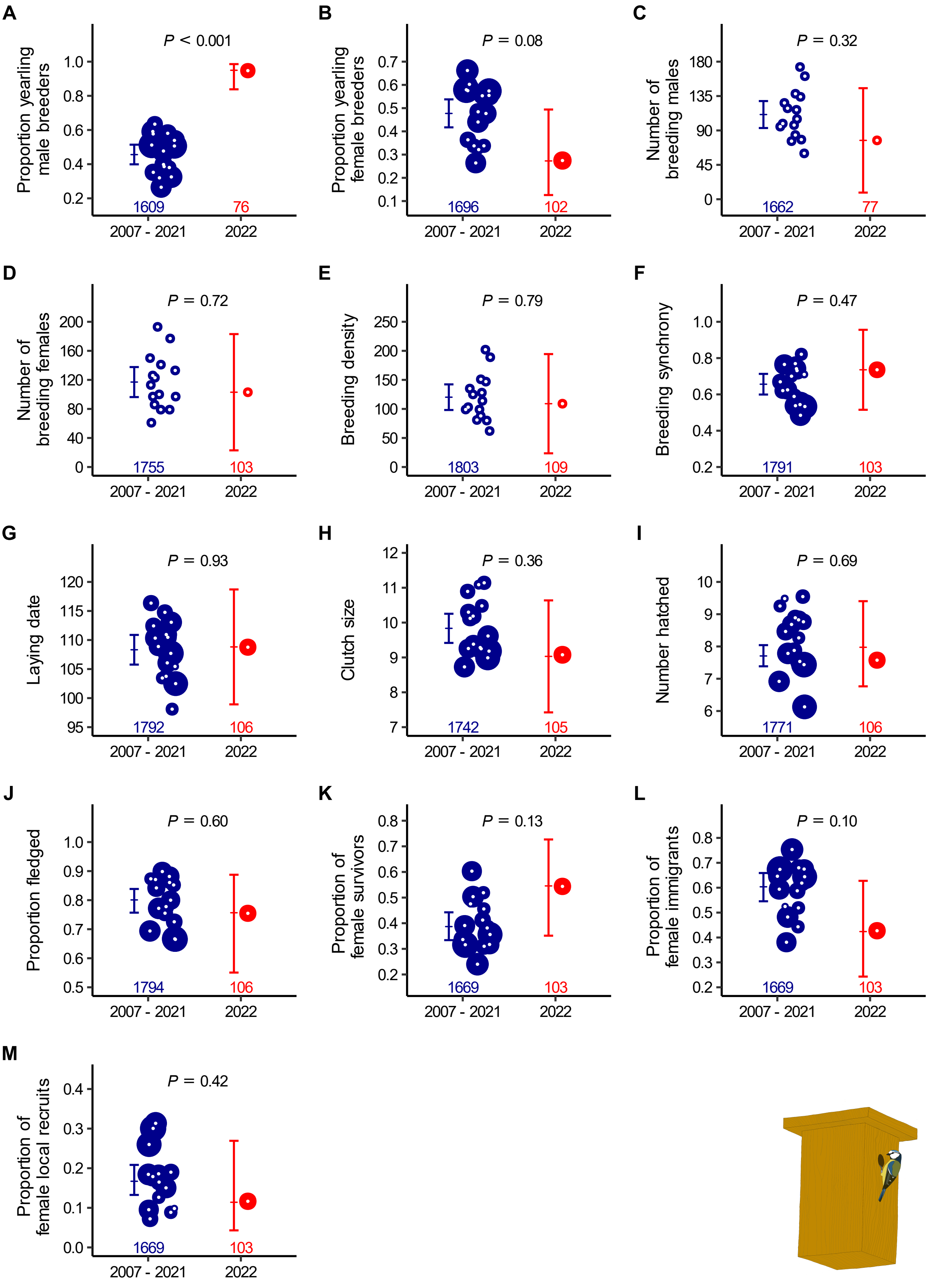

Fig A. Comparison of key breeding parameters between the experimental year 2022 (red) and the control years 2007 – 2021 (blue).

(A) Proportion of yearlings among breeding males.
(B) Proportion of yearlings among breeding females.
(C) Number of breeding males.
(D) Number of breeding females.
(E) Breeding density (number of breeding pairs, i.e. unique male-female combinations).
(F) Breeding synchrony (synchrony index [S3]).
(G) Laying date (day of year, 1 = 1 January).
(H) Clutch size.
(I) Number of hatchlings.
(J) Proportion fledged (proportion of nests that produced at least one fledgling).
(K) Proportion of breeding females that were breeding in previous season.
(L) Proportion of breeding females first recorded in present season.
(M) Proportion of breeding females that hatched from a nest on the study site in previous season.
Shown are annual raw data (dots, size varies according to sample size, annual means or proportions) and model fits with their 95% confidence intervals (bars or fitted lines with shaded areas). Numbers at the bottom indicate overall sample sizes. Sample sizes differ for (K), because age information is not available for all breeding females. See Table S3 for statistical details. The models for A, B, J–M have a binary response variable (Y/N). The data and code needed to generate this Figure can be found in https://osf.io/w7fx6.

SUPPORTING REFERENCES

S1. Schlicht E, Gilsenan C, Santema P, Türk A, Wittenzellner A, Kempenaers B. Data and analytical code for "Removal of older males increases extra-pair siring success of yearling males." 2024. https://osf.io/w7fx6.

S2. Schlicht E, Kempenaers B. Age trajectories in extra- pair siring success suggest an effect of maturation or early- life experience. J Evol Bio. 2023; 36: 1213–1225. doi:10.1111/jeb.14201

S3. Kempenaers B. The use of a breeding synchrony index. Ornis Scand. 1993;24: 84. doi: 10.2307/3676415.

S4. Brouwer L, Griffith SC. Extra-pair paternity in birds. Mol Ecol. 2019;28: 4864–4882. doi:10.1111/mec.15259
